# Association of glymphatic system dysfunction with cognitive impairment in temporal lobe epilepsy

**DOI:** 10.3389/fnagi.2024.1459580

**Published:** 2024-10-18

**Authors:** Jiajia Wang, Xiaona Xia, Bin Zhang, Xiaotian Ma, Feng Shi, Ying Wei, Ling Li, Xiangshui Meng

**Affiliations:** ^1^Department of Radiology, Qilu Hospital of Shandong University, Jinan, China; ^2^Department of Radiology, Qilu Hospital (Qingdao), Cheeloo College of Medicine, Shandong University, Qingdao, China; ^3^Department of Neurology, Qilu Hospital (Qingdao), Cheeloo College of Medicine, Shandong University, Qingdao, China; ^4^Department of Medicine Experimental Center, Qilu Hospital (Qingdao), Cheeloo College of Medicine, Shandong University, Qingdao, China; ^5^Department of Research and Development, Shanghai United Imaging Intelligence Co., Ltd., Shanghai, China

**Keywords:** central nervous system, temporal lobe epilepsy, diffusion tensor imaging, glymphatic system, semantic fluency, choroid plexus

## Abstract

**Objectives:**

To explore the relationship between glymphatic dysfunction and cognitive impairment in unilateral temporal lobe epilepsy (TLE).

**Methods:**

This study retrospectively included 38 patients with unilateral TLE and 26 age- and gender-matched healthy controls (HCs). The diffusion tensor image analysis along the perivascular space (DTI-ALPS) index, choroid plexus volume (CPV), and cognitive assessment were obtained for each participant. Neuropsychological test batteries included Montreal Cognitive Assessment (MoCA), Minimum Mental State Examination, Arithmetic Test (AT), Digit Symbol Substitution Test (DSST), Digit Span Test (DST), Boston Naming Test, Block design, Phonological Fluency Test (PFT), and Semantic Verbal Fluency (SVF).

**Results:**

Compared to HCs, TLE patients had lower scores of MoCA, AT, DSST, DST, Block design, PFT and SVF (all *p* < 0.05) and lower values of mean DTI-ALPS index (1.491 ± 0.142 vs. 1.642 ± 0.123, *p* < 0.001). Significantly lower DTI-ALPS index values were observed in the ipsilateral hemisphere than in the contralateral hemisphere (1.466 ± 0.129 vs. 1.517 ± 0.175, *p* = 0.013) for patients with unilateral TLE. Correlation analyses found that SVF performance was significantly or borderline significantly associated with glymphatic function (*FDR-corrected p* < 0.05 for all DTI-ALPS index and *FDR-corrected p* = 0.057 for CPV) in TLE patients. Linear regression analyses showed that increased CPV and decreased DTI-ALPS index were independent risk factors for semantic fluency impairment (all *p* < 0.05). Furthermore, mediation analyses found the mediator role of the mean DTI-ALPS index in the relationship between choroid plexus enlargement and semantic fluency impairment (indirect effect: *β* = −0.182, *95%CI* = −0.486 to −0.037).

**Conclusion:**

These findings reveal the important role of the DTI-ALPS index and CPV in SVF performance in unilateral TLE. Decreased DTI-ALPS index and increased CPV are the independent risk factors for semantic fluency impairment. The DTI-ALPS index may fully mediate the relationship between CP enlargement and SVF performance. These insights provide a radiological foundation for further investigations into the mechanism of the glymphatic system in TLE pathophysiology.

## 1 Introduction

Over 70 million individuals worldwide suffer from epilepsy, a serious neurological condition of which temporal lobe epilepsy (TLE) accounts for 30–35% (Hauser et al., 1996; Thijs et al., 2019; Beghi, 2020). Approximately 30–40% of adult patients with epilepsy suffer from cognitive problems, with language, verbal memory, executive function, and attention being the most vulnerable domains (Wang et al., 2020), which has an important influence on quality of life and cognitive rehabilitation may help improve quality of life (Meneses et al., 2009).

The glymphatic system (GS) is a vital way to remove metabolites and misfolded peptides/proteins from the brain (Iliff et al., 2012). A novel non-invasive technique named diffusion tensor image analysis along the perivascular space (DTI-ALPS) has proven useful for evaluating glymphatic function in epilepsy (Lee D. A. et al., 2022), a condition often links to GS dysfunction (Zhang et al., 2023; Zhao et al., 2023; Hlauschek et al., 2024). Several studies have demonstrated the potential of the DTI-ALPS in localizing epileptogenic foci, evaluating surgical outcomes in unilateral TLE (Zhang et al., 2023), and providing insights into the diagnosis of childhood absence epilepsy (Pu et al., 2023). Despite some controversies surrounding this index raised in recent years (Piantino et al., 2023; Ringstad, 2024), previous studies have demonstrated that the DTI-ALPS index is consistent with other GS evaluation methods, such as glymphatic MRI, which is considered the gold standard for MRI assessment of GS using gadolinium-based contrast agents as intrathecal tracers (Zhang W. et al., 2021). Therefore, we have reason to believe that this index may serve as a potential imaging biomarker reflecting GS function. Additionally, enlarged choroid plexus (CP), an important GS component, has been associated with impaired glymphatic clearance according to a recent glymphatic MRI study (Li et al., 2023). Unlike the DTI-ALPS index, less attention has been paid to CP, even though it is one of the important components of GS (Christensen et al., 2022).

During normal aging, GS plays a protective role in cognitive decline (Wang et al., 2023). The relationship between GS function and cognitive performance has been reported across various central nervous system disorders (Taoka et al., 2017; Chen et al., 2021; Zhang Y. et al., 2021). For instance, lower DTI-ALPS index has been correlated with worser performance on tests like the Boston Naming Test (BNT), Trail Making Test A, and Digit Span Test (DST) in Alzheimer’s disease (AD) (Zhang et al., 2024). Mediation analysis further suggested that cognitive impairment in AD may be significantly mediated by GS dysfunction (Hsu et al., 2023). Similar associations between global cognition and regional glymphatic function have been observed in behavioral variant frontotemporal dementia (Jiang et al., 2023a). However, the relationship between glymphatic function and cognitive performance in unilateral TLE patients remains an understudied area.

Thus, in this study for patients with unilateral TLE, we mainly aimed to investigate (1) whether the DTI-ALPS index, in the ipsilateral hemisphere is reduced compared to the contralateral hemisphere; (2) the association between the ALPS index, CPV and performance on a battery of neuropsychological tests; and (3) whether the DTI-ALPS index mediates the relationship between CP enlargement and specific neuropsychological performance.

## 2 Materials and methods

This study followed the ethical guidelines expressed in the Declaration of Helsinki and the study protocol was approved by the ethics committee of Qilu Hospital of Shandong University (Qingdao) (KYLL-qdql2020070). Informed consent was exempt due to the retrospective nature of our study.

### 2.1 Participants

**Figure 1:**
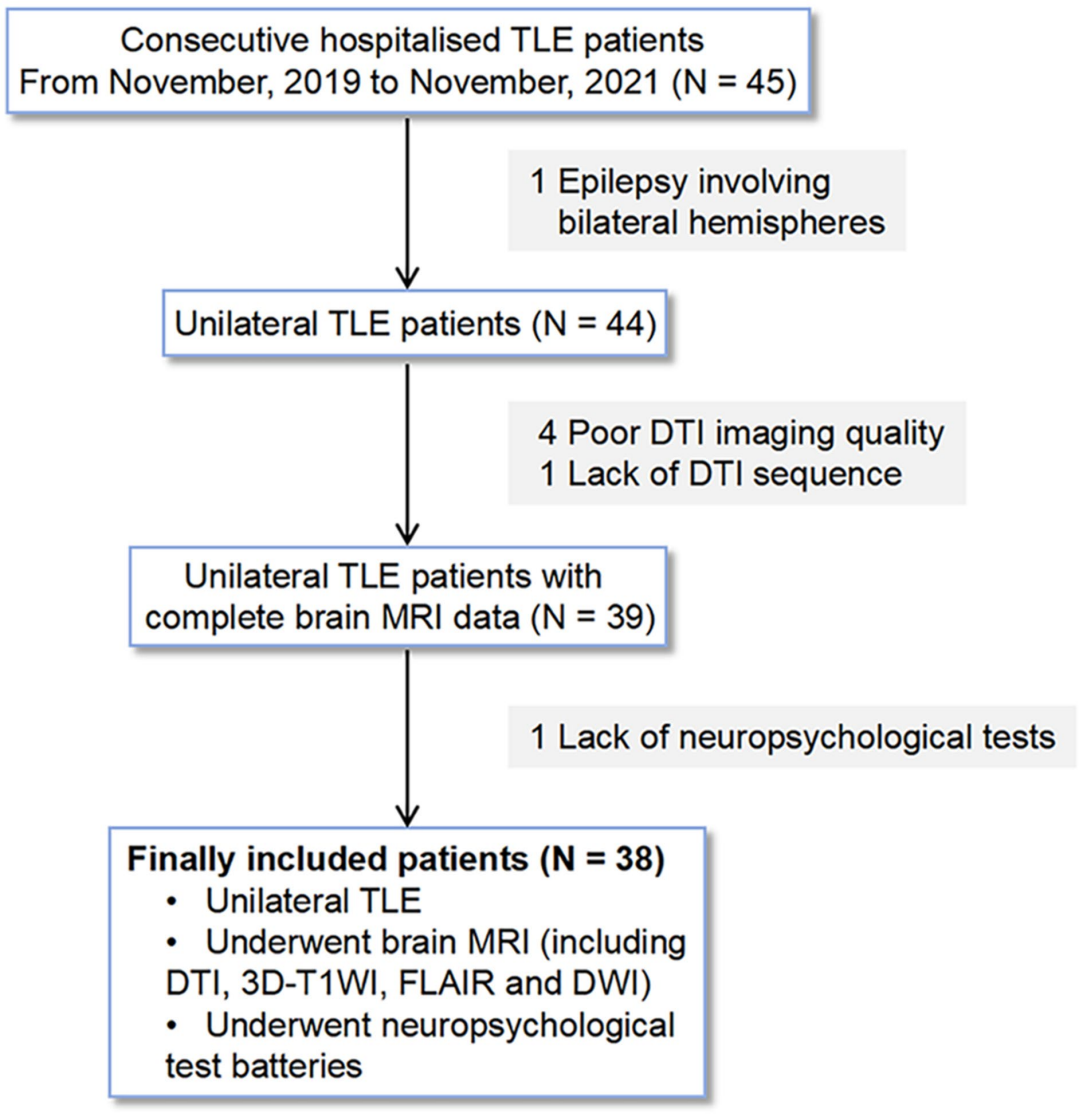
Selection process for patients with unilateral TLE in this study.

We analyzed consecutive hospitalized patients with unilateral TLE in the Neurology Department at our hospital from November, 2019 to November, 2021 based on the following inclusion criteria: (1) clinical diagnosis of unilateral TLE based on video-electroencephalography telemetry, seizure semiology, and neuroimaging by epileptologists (Stasenko et al., 2023); (2) brain MRI scans were performed, including diffusion tensor imaging (DTI), three-dimensional T1-weighted imaging (3D-T1WI), fluid-attenuated inversion recovery (FLAIR), and diffusion weighted imaging (DWI); (3) underwent a battery of neuropsychological tests within 1 week before the MRI examination. Patients were excluded if they met the following criteria: (1) brain parenchymal lesions such as tumors and vascular malformations confirmed by MRI; (2) sleep deprivation in the week before the MRI scans; (3) poor DTI image quality for analyses of glymphatic function; (4) a history of neuropsychiatric disorders; (5) a history of neurodegenerative diseases, such as AD and Parkinson’s disease; (6) epilepsy involving bilateral hemispheres. Of the 45 patients with TLE, four patients with poor DTI image quality, one without DTI data, one with epilepsy involving bilateral hemispheres, and one without neuropsychological data were excluded. Thus 38 patients with unilateral TLE were retrospectively selected in this study and divided into left TLE or right TLE groups according to their clinical symptoms, video electroencephalography, and neuroimaging findings. The detailed selection process is shown in Figure 1. Inclusion criteria for healthy controls (HCs) were as follows: (1) brain MRI scans including DTI, 3D-T1WI, FLAIR and DWI; (2) underwent a battery of neuropsychological tests within 1 week before the MRI examination. And the exclusion criteria were as follows: (1) brain parenchymal lesions; (2) sleep deprivation in the week before the MRI scans; (3) a history of neuropsychiatric disorders; (4) a history of neurodegenerative diseases, such as AD and Parkinson’s disease; (5) a history of epilepsy. Ultimately, 26 age- and gender-matched HCs were included in the study.

### 2.2 Clinical assessments

The following basic clinical information was collected: age, gender, education level, age of first onset of seizure, disease duration of epilepsy, seizure time, seizure frequency, sleep time per day, and Pittsburgh Sleep Quality Index. Additionally, neuropsychological assessments were performed including the Montreal Cognitive Assessment (MoCA), Minimum Mental State Examination (MMSE), Arithmetic Test (AT), Digit Symbol Substitution Test (DSST), DST, BNT, Block design, Phonological Fluency Test (PFT), and Semantic Verbal Fluency (SVF).

### 2.3 Image acquisition

All participants underwent MRI scans using the same protocol on a single 3 T MRI scanner (Ingenia, Philips Medical Systems, Netherlands) equipped with an eight-channel brain phased-array coil. No software or hardware updates were made to the MRI scanner during the entire scan period for the participants to ensure the consistency of the collected image data. The parameters of the MR sequences were as follows:

1. 3D-T1WI was acquired using a fast spoiled gradient echo sequence: repetition time (TR) = 6.7 msec, echo time (TE) = 3.0 msec, flip angle (FA) = 8°, field of view (FOV) = 240 mm × 240 mm, data matrix = 240 × 240, slice thickness (ST) = 1 mm, gap = 0 mm, 170 slices, number of signals averaged (NSA) = 1, orientation: sagittal.
2. T2-FLAIR: TR = 7,000 msec, TE = 125 msec, FA = 90°, FOV = 230 mm × 230 mm, data matrix = 288 × 163, ST = 6 mm, gap = 1 mm, 18 slices, NSA = 1, orientation: transverse.
3. DWI: TR = 2,235 msec, TE = 76 msec, FA = 90°, FOV = 230 mm × 230 mm, data matrix = 176 × 134, ST = 6 mm, gap = 1 mm, 18 slices, NSA = 1, orientation: transverse.
4. DTI was acquired using echo planar imaging with a total of 32 different diffusion directions with b = 1,000 s/mm^2^ and 1 non-diffusion-weighted (b = 0) image: TR = 4,900 msec, TE = 95 msec, FA = 90°, FOV = 224 mm × 224 mm, data matrix = 112 × 110, ST =2 mm, gap = 0 mm, 70 slices, NSA = 2, orientation: transverse.

### 2.4 Image preprocessing for brain volume

With 3D-T1WI, an image analysis tool named uRP, developed by Shanghai United Imaging Intelligence Co. Ltd., was used for brain segmentation and volume acquisition (Wu et al., 2023). The uRP facilitated a comprehensive preprocessing pipeline that included skull stripping, bias field correction, and image resampling to attain an isotropic resolution of 1 × 1 × 1 mm^3^. Subsequently, bilateral choroid plexus volume (CPV) of the lateral ventricles and total intracranial volume (ICV) were quantified according to the Desikan-Killiany atlas using a pre-trained cascaded VB-Nets model integrated into the uRP tool. The VB-Nets model is capable of segmenting the Desikan-Killiany atlas with an average Dice similarity coefficient of 91.06% between the automatic segmentation and Freesurfer-based segmentation (Ge et al., 2022). The CPV for each participant was calculated by summing the values of the left and right CPV. For unilateral TLE patients, CPV of both the ipsilateral and contralateral hemispheres were also used in the analyses. Examples of CP segmentation are shown in Supplementary Figure S1. To account for individual heterogeneity in ICV, CPV was presented as a percentage of ICV (CPV/ICV) as previously proposed (Choi et al., 2022).

### 2.5 Preprocessing of DTI data

DSI Studio software (version 2023 Aug) was used to preprocess the DTI data. The key steps were: (i) converted the original Digital Imaging and Communications in Medicine data into the SRC format; (ii) performed quality control to identify and address issues like eddy current artifacts or head motion (Yeh et al., 2019); (iii) preprocessing including eddy current correction, motion correction, and skull stripping; (iv) reconstruction using the DTI method (Yeh et al., 2013; Tax et al., 2022).

### 2.6 Calculation of the DTI-ALPS index

A 4 × 4 × 4 mm^3^ region of interest (ROI) was placed on the projection and association fibers at the level of the lateral ventricle body in each hemisphere, respectively. The diffusivity along the x-, y- and z-axis was obtained at the voxel level of ROI. The DTI-ALPS index was subsequently calculated using the formula (Lee H. J. et al., 2022):


$$DTI-\mathrm{ALPS}\;\mathrm{index}=\frac{\mathrm{mean}\;\left(\mathrm{Dxxproj,Dxxassoc}\right)}{\mathrm{mean}\;\left(\mathrm{Dyyproj,Dzzassoc}\right)}$$

Where Dxxproj and Dxxassoc are the diffusivity along the x-axis in the projection fiber and association fiber, respectively. Dyyproj means the diffusivity along the y-axis in the projection fiber, and Dzzassoc means the diffusivity along the z-axis in the association fiber. This was done for all participants by an experienced neuroradiologist (XSM, with more than 20 years of experience) blinded to clinical data. In a subset of 30 participants, another neuroradiologist (XNX, with 10 years of experience) repositioned the ROIs and then calculated the DTI-ALPS index to evaluate the inter-rater reliability. The mean DTI-ALPS index for each participant was the average of bilateral values. For TLE patients, the DTI-ALPS index obtained from both the ipsilateral hemisphere and contralateral hemisphere was used in our analyses.

### 2.7 Statistical analyses

Categorical data are shown as frequencies (percentages) and continuous data are presented as mean ± standard deviation or median (interquartile range) based on distribution. The intraclass correlation coefficient (two-way mixed model, single measure, absolute agreement) was used to assess the inter-rater reliability of the DTI-ALPS index. Baseline differences between groups (TLE vs. HCs, left TLE vs. right TLE) were analyzed using two-sample *t*-test or Mann–Whitney test for continuous variables, and chi-square test or Fisher’s exact test for categorical variables when appropriate.

Paired *t*-test was used to investigate whether the ipsilateral DTI-ALPS index or CPV/ICV differed from the contralateral side. Pearson’s correlation or Spearman’s rank correlation analyses were performed to test associations, and correlations were expressed as Pearson’s correlation coefficient (r) or Spearman’s correlation coefficient (*ρ*). To address the issue of multiple comparisons, the Benjamini-Hochberg false discovery rate (FDR) correction was applied to *p*-values. *FDR-corrected* *p* < 0.05 is considered statistically significant. To identify independent risk or protective factors for neuropsychological performance, the multivariable stepwise linear regression analysis with backward elimination method (*p* ≥ 0.10 for exclusion) was constructed using baseline variables that were clinically relevant or showed a univariate association (*p* ≤ 0.10) with neuropsychological scores. The significance threshold was set at *p* < 0.05.

Exploratory analyses were conducted to investigate whether the DTI-ALPS index mediated the relationship between CP enlargement and neuropsychological performance. The “mediation” and “BruceR” packages (Tingley et al., 2014; Bao, 2023) were used to evaluate the direct and indirect (mediation) effects. The 95% confidence intervals (CI) and standard errors for the total/direct/indirect effects were acquired by random sampling (set to 1,000) with replacement, a reliable non-parametric technique for CI construction called bootstrapping (Alfons et al., 2022). We primarily investigated whether the mean DTI-ALPS index mediates the relationship between CPV and SVF performance. Besides, we also explored the mediation role of ipsilateral (or contralateral) DTI-ALPS index on the relationship between ipsilateral (or contralateral) CPV and SVF performance. A significant effect is indicated if the bootstrapped 95% CI does not include zero (Osborne et al., 2023).

The mediation analyses were performed using the R statistical program (version 4.2.3), while all other statistical analyses were conducted using SPSS (version 26).

## 3 Results

### 3.1 Clinical, neuropsychological, and imaging characteristics

**Figure 2:**
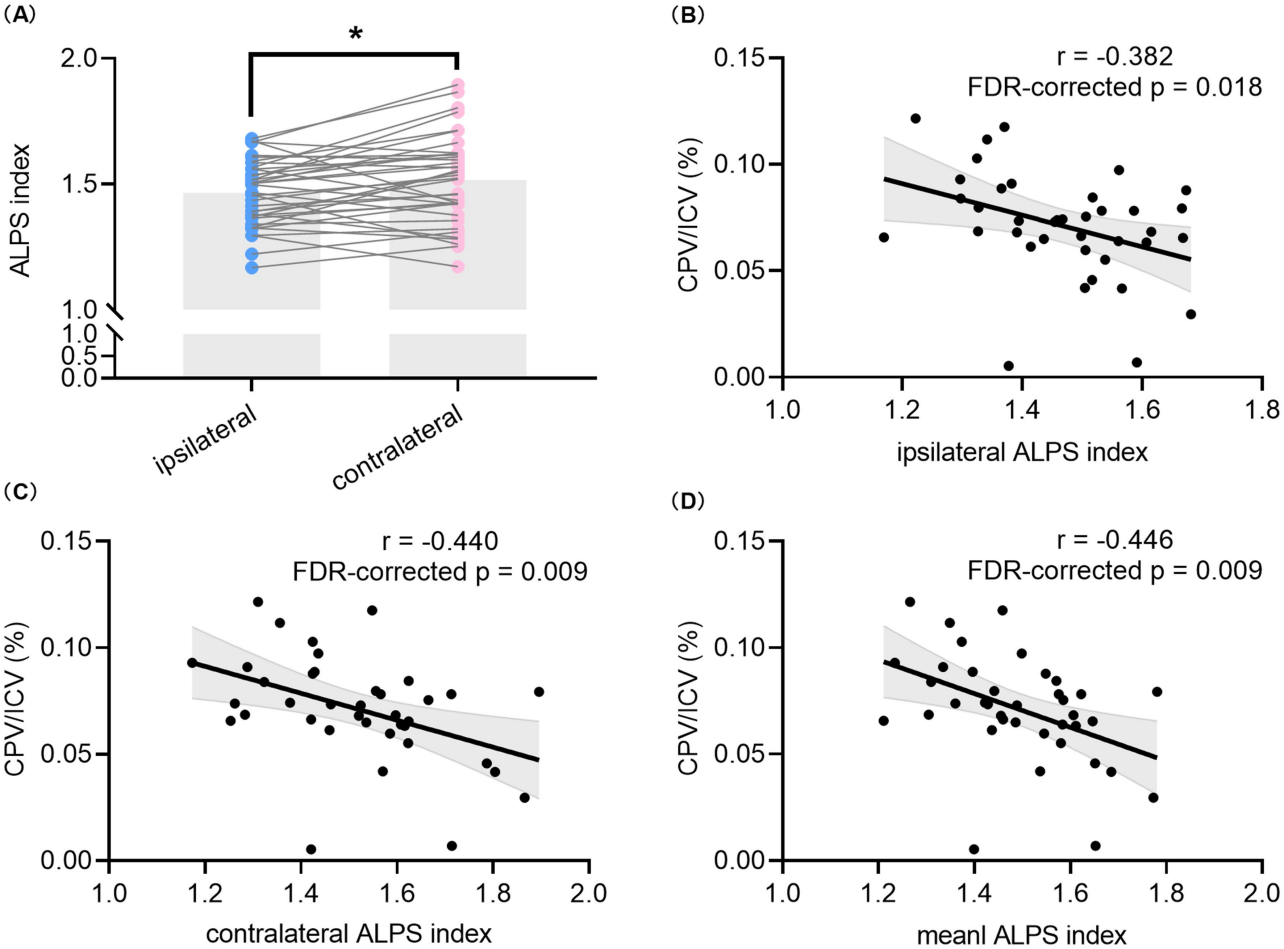
Comparison of the DTI-ALPS index between hemispheres and correlation analyses between the DTI-ALPS index and CPV in all TLE patients. Comparison between the ipsilateral DTI-ALPS index and contralateral DTI-ALPS index in patients with unilateral TLE **(A)**. Correlation between CPV/ICV and the ipsilateral DTI-ALPS index **(B)** as well as contralateral DTI-ALPS index **(C)**. Correlation between the mean DTI-ALPS index of bilateral hemispheres and CPV/ICV **(D)**. * indicates *p* < 0.05. The *p* values with FDR correction of correlation analyses are shown in the figure. TLE, temporal lobe epilepsy; DTI, diffusion tensor imaging; ALPS, analysis along the perivascular space; CPV, choroid plexus volume; ICV, intracranial volume; FDR, false discovery rate.

Table 1 summarizes the clinical, neuropsychological, and imaging characteristics of all participants. The intraclass correlation coefficient of the DTI-ALPS index was 0.879 (*95% CI*: 0.805–0.926), which showed the excellent repeatability between two observers. The HCs were age- and gender-matched with unilateral TLE patients (*p* = 0.256 and 0.690), and no significant differences in ICV or CPV/ICV were found between the two groups. Compared to HCs, TLE patients had lower scores of MoCA, AT, DSST, DST, Block design, PFT and SVF (all *p* < 0.05) and lower values of mean DTI-ALPS index (1.491 ± 0.142 vs. 1.642 ± 0.123, *p* < 0.001). Paired t-test in all unilateral TLE patients found that the ipsilateral DTI-ALPS index was significantly lower than that in the contralateral side (1.466 ± 0.129 vs. 1.517 ± 0.175, *p* = 0.013) (Figure 2A), but CPV/ICV was not (0.035 ± 0.013 vs. 0.036 ± 0.014, *p* = 0.577). In addition, there were no significant differences in any of the characteristics between patients with left TLE and right TLE.

**Table 1:** Clinical, neuropsychological and imaging characteristics in patients with TLE and HCs.

Characteristics	TLE			HCs (*N* = 26)	*p* value^a^
	Total (*N* = 38)	Left (*N* = 19)	Right (*N* = 19)		
Clinical characteristics					
Age (years)	32.0 (21.0–50.3)	32.0 (24.0–54.0)	30.0 (21.0–49.0)	23.0 (21.8–34.0)	0.256
Gender: male	20 (52.6%)	9 (47.4%)	11 (57.9%)	15 (57.7%)	0.690
Education level					
Less than college	20 (52.6%)	7 (36.8%)	13 (68.4%)	14 (53.8%)	0.924
College or equivalent	18 (47.4%)	12 (63.2%)	6 (31.6%)	12 (46.2%)	
Sleep time per day (hours)	8.0 (6.8–8.1)	8.0 (6.0–8.0)	7.5 (7.0–9.0)	7.0 (7.0–8.0)	0.218
Pittsburgh sleep quality index	3.0 (2.0–6.0)	3.0 (2.0–7.0)	3.0 (2.0–5.0)	4.0 (2.0–5.3)	0.756
Age of first onset of seizure				NA	
<18 years	10 (26.3%)	4 (21.1%)	6 (31.6%)		
≥18 years	28 (73.7%)	15 (78.9%)	13 (68.4%)		
Disease duration of epilepsy (months)	36.0 (12.5–72.0)	36.0 (10.0–52.0)	60.0 (18.0–121.0)	NA	
Seizure time				NA	
Only awake	21 (55.3%)	10 (52.6%)	11 (57.9%)		
Only sleep	6 (15.8%)	4 (21.1%)	2 (10.5%)		
Awake and sleep	11 (28.9%)	5 (26.3%)	6 (31.6%)		
Seizure frequency				NA	
0-11/Year	20 (52.6%)	10 (52.6%)	10 (52.6%)		
≥11/Year	18 (47.4%)	9 (47.4%)	9 (47.4%)		
Neuropsychological assessments					
MoCA	27.5 (23.8–29.0)	29.0 (24.0–30.0)	27.0 (23.0–29.0)	29.0 (28.0–30.0)	0.003
MMSE	30.0 (29.0–30.0)	30.0 (29.0–30.0)	30.0 (27.0–30.0)	29.0 (28.8–30.0)	0.447
AT	11.0 (7.8–13.3)	12.0 (6.0–14.0)	9.0 (8.0–13.0)	16.0 (14.8–17.3)	<0.001
DSST	58.5 (45.8–67.5)	61.0 (40.0–72.0)	53.0 (46.0–65.0)	72.0 (64.8–75.0)	<0.001
DST	4.0 (3.0–7.0)	5.0 (3.0–7.3)	4.0 (3.0–7.0)	8.0 (6.8–8.0)	<0.001
BNT	27.0 (23.5–29.0)	26.5 (20.8–29.0)	27.0 (24.0–29.0)	27.0 (26.8–28.0)	0.355
Block design	33.0 (27.5–41.5)	36.0 (29.0–42.0)	28.5 (25.8–39.5)	45.0 (42.5–47.5)	<0.001
PFT	9.4 ± 6.0	10.7 ± 6.5	8.1 ± 5.3	20.7 ± 6.0	<0.001
SVF	13.3 ± 4.2	13.4 ± 4.3	13.2 ± 4.1	17.9 ± 5.0	<0.001
Imaging characteristics					
ICV (cm^3^)	1429.8 ± 133.9	1415.8 ± 123.2	1443.9 ± 145.9	1462.4 ± 129.7	0.336
CPV/ICV (%)	0.071 ± 0.025	0.075 ± 0.024	0.068 ± 0.026	0.068 ± 0.016	0.529
Ipsilateral CPV/ICV (%)	0.037 (0.030–0.039)	0.037 (0.030–0.0.39)	0.036 (0.028–0.039)	NA	
Contralateral CPV/ICV (%)	0.036 ± 0.014	0.039 ± 0.013	0.033 ± 0.014	NA	
Mean DTI-ALPS index	1.491 ± 0.142	1.495 ± 0.161	1.487 ± 0.123	1.642 ± 0.123	<0.001
Ipsilateral DTI-ALPS index	1.466 ± 0.129	1.466 ± 0.146	1.466 ± 0.115	NA	
Contralateral DTI-ALPS index	1.517 ± 0.175	1.525 ± 0.190	1.509 ± 0.164	NA	

Categorical variables are shown as frequencies (percentages) and continuous variables are presented as mean ± standard deviation or median (interquartile range) in accordance with their distribution.

a Represents the comparison of the characteristics between the TLE group (*N* = 38) and the HCs group (*N* = 26), *p* < 0.05 indicates statistically significant.

TLE, temporal lobe epilepsy; HCs, healthy controls; MoCA, Montreal Cognitive Assessment; MMSE, Minimum Mental State Examination; AT, Arithmetic Test; DSST, Digit Symbol Substitution Test; DST, digital span test; BNT, Boston Naming Test; PFT, Phonological Fluency Test; SVF, Semantic Verbal Fluency; ICV, intracranial volume; CPV, choroid plexus volume; DTI-ALPS, diffusion tensor image analysis along the perivascular space; NA, not available.

### 3.2 Correlation analyses between the DTI-ALPS index and CPV

As shown in Figures 2B–D, significant negative correlations were found between CPV/ICV and the ipsilateral DTI-ALPS index (*r* = −0.382, *p* = 0.018, *FDR-corrected p* = 0.018), the contralateral DTI-ALPS index (*r* = −0.440, *p* = 0.006, *FDR-corrected p* = 0.009), and the mean DTI-ALPS index (*r* = −0.446, *p* = 0.005, *FDR-corrected p* = 0.009) in all TLE patients. Furthermore, CPV was always correlated with the corresponding DTI-ALPS index in all TLE patients, regardless of whether it was contralateral or ipsilateral, as shown in Supplementary Figure S2. However, no significant correlation was found between the mean DTI-ALPS index and CPV/ICV in the HCs group (data not shown).

### 3.3 Correlation analyses between the DTI-ALPS index, CPV and neuropsychological performance

**Figure 3:**
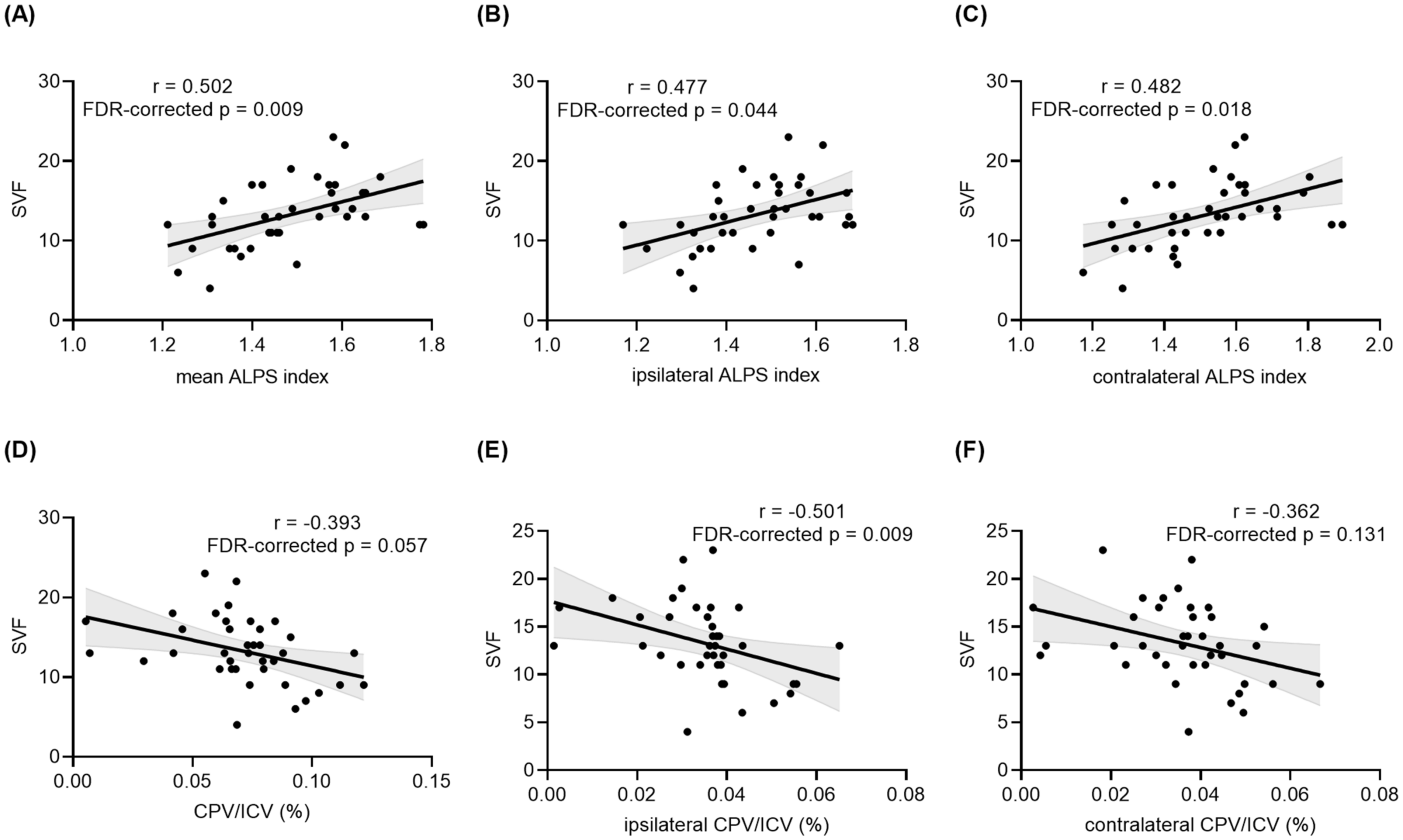
Correlation analyses between the DTI-ALPS index, CPV and SVF performance in all TLE patients. Correlation between SVF performance and the mean ALPS index of bilateral hemispheres **(A)**, ipsilateral ALPS index **(B)**, contralateral ALPS index **(C)**, CPV/ICV **(D)**, ipsilateral CPV/ICV **(E)**, and contralateral CPV/ICV **(F)** in patients with unilateral TLE. The *p* values with FDR correction are shown in the figure. TLE, temporal lobe epilepsy; DTI, diffusion tensor imaging; ALPS, analysis along the perivascular space; CPV, choroid plexus volume; ICV, intracranial volume; SVF, Semantic Verbal Fluency; FDR, false discovery rate.

Among neuropsychological tests, the ipsilateral DTI-ALPS index showed a significantly moderate positive correlation with SVF scores (*r* = 0.477, *uncorrected p* = 0.0049 and *FDR-corrected p* = 0.044) in patients with TLE. Positive correlations were also discovered between SVF scores and the contralateral DTI-ALPS index (*r* = 0.482, *uncorrected p* = 0.002, and *FDR-corrected p* = 0.018) as well as the mean DTI-ALPS index (*r* = 0.502, *uncorrected p* = 0.001, *FDR-corrected p* = 0.009) for TLE patients. A borderline significant correlation was found between CP enlargement and AT, DSST and SVF scores (*r* = −0.400, −0.380, −0.393, *uncorrected p* = 0.013, 0.019, 0.015, *FDR-corrected p* = 0.057, 0.057, 0.057, respectively) in the TLE group. Interestingly, SVF was negatively correlated with ipsilateral CPV/ICV (*r* = −0.501, *uncorrected p* = 0.001 and *FDR-corrected p* = 0.009) but not with contralateral CPV/ICV (*r* = −0.362, *uncorrected p* = 0.026 and *FDR-corrected p* = 0.131) in patients with TLE. Details for correlation analyses for patients with TLE are shown in Figure 3 and Supplementary Table S1. Additional correlation analyses in the HCs group showed that CPV/ICV was only negatively correlated with MMSE scores (Supplementary Table S2).

### 3.4 Linear regression analyses to explore the role of the DTI-ALPS index and CPV in semantic fluency performance in the TLE group

Multivariable stepwise linear regression analyses showed that the ipsilateral DTI-ALPS index (*β* = 0.393, *p* = 0.008) was a significant independent protective factor for SVF scores, after adjusting for the potential confounder in Model 1, as shown in Table 2. The contralateral DTI-ALPS index and mean DTI-ALPS index were also significant independent predictors for SVF scores (*β* = 0.503, 0.487 and *p* = 0.0004, 0.001 in Model 2, Model 3, respectively). Conversely, CP enlargement was identified as an independent risk factor for SVF performance (*β* = −0.330, *p* = 0.03 in Model 4), which was likewise found in both contralateral and ipsilateral CPV (Supplementary Table S3).

**Table 2:** Final results of multivariable linear regression (backward stepwise) to explore the role of the DTI-ALPS index and CPV in semantic fluency performance in the TLE group

		B	Beta coefficient (*β*)	*p* value	Adjust-R^^2^^
Model 1	Educational level^a^				0.285
	College or equivalent	2.924	0.356	0.016	
	Ipsilateral DTI-ALPS index	12.619	0.393	0.008	
Model 2	Educational level^a^				0.392
	College or equivalent	3.606	0.440	0.002	
	Contralateral DTI- ALPS index	11.938	0.503	<0.001	
Model 3	Educational level^a^				0.375
	College or equivalent	3.253	0.397	0.004	
	Mean DTI-ALPS index	14.252	0.487	0.001	
Model 4	Educational level^a^				0.236
	College or equivalent	2.920	0.356	0.02	
	CPV/ICV (%)	-54.380	-0.330	0.03	

The initial regression model included the following six variables: age, sex, education level (divided into less than college and college or equivalent), disease duration, seizure frequency (divided into 0-11/Year and ≥11/Year), ipsilateral ALPS index (Model 1) or contralateral ALPS index (Model 2) or mean ALPS index (Model 3) or CPV/ICV (Model 4). Using the backward elimination method, models were ultimately constructed by the above variables in the table, which indicated that the decreased DTI-ALPS index and increased CPV/ICV were independent risk factors for semantic fluency impairment. This table shows the variables entered into the final model after backward elimination. Variables not included in the final model (age, sex, disease duration and seizure frequency) are not shown.

^aReference category: less than college.^

SVF, Semantic Verbal Fluency; TLE, Temporal lobe epilepsy; DTI-ALPS, diffusion tensor image analysis along the perivascular space; CPV, choroid plexus volume; ICV, intracranial volume.

### 3.5 Exploratory mediation analyses to investigate the role of the DTI-ALPS index in semantic fluency performance in the TLE group

Mediation analyses demonstrated that the indirect effect of CPV on SVF performance through the mean DTI-ALPS index was statistically significant (*β* = −0.182, *95%CI* = −0.486 to −0.037), whereas the direct effect was not (*β* = −0.212, *95%CI* = −0.540 to 0.153). Likewise, the indirect effects of contralateral CPV on SVF performance through the contralateral DTI-ALPS index (*β* = −0.179, *95%CI* = −0.461 to −0.031), as well as those of ipsilateral CPV on SVF performance through the ipsilateral DTI-ALPS index (*β* = −0.120, *95%CI =* −0.354 to −0.006), were also found to be significant. Details are shown in Table 3.

**Table 3:** Simple mediation analyses of the DTI-ALPS index in the relationship between CP enlargement and SVF performance in all TLE patients.

	*β*	SE	*95%CI*
Predictor (X): CPV/ICV; Mediators (M): mean ALPS; Outcome (Y): SVF			
Total effect	−0.393	0.141	[−0.731, −0.174]
Indirect effect	−0.182	0.120	[−0.486, −0.037]
Direct effect	−0.212	0.171	[−0.540, 0.153]
Predictor (X): ipsilateral CPV/ICV; Mediators (M): ipsilateral ALPS; Outcome (Y): SVF			
Total effect	−0.387	0.144	[−0.725, −0.167]
Indirect effect	−0.120	0.091	[−0.354, −0.006]
Direct effect	−0.267	0.164	[−0.610, 0.046]
Predictor (X): contralateral CPV/ICV; Mediators (M): contralateral ALPS; Outcome (Y): SVF			
Total effect	−0.362	0.149	[−0.684, −0.117]
Indirect effect	−0.179	0.113	[−0.461, −0.031]
Direct effect	−0.183	0.174	[−0.493, 0.180]

SE and CI are estimated based on 1,000 bootstrap samples. The presence of a non-zero bootstrapped 95% CI indicates a significant effect.

DTI-ALPS, diffusion tensor image analysis along the perivascular space; CPV, choroid plexus volume; ICV, intracranial volume; SVF, Semantic Verbal Fluency; SE, standard error; CI, confidence interval.

## 4 Discussion

This study represents the first attempt to investigate the role of glymphatic function on SVF performance in unilateral TLE patients using non-invasive 3D-TIWI and DTI methods. The main findings of the present study in patients with unilateral TLE were as follows: (1) the ipsilateral side exhibited decreased glymphatic clearance function compared to the contralateral side, as indicated by the DTI-ALPS index; (2) both the increased CPV/ICV and decreased DTI-ALPS index, which significantly correlated with each other, were identified as independent risk factors for semantic fluency impairment; and (3) the DTI-ALPS index may totally mediate the relationship between CP enlargement and semantic fluency impairment.

GS plays a crucial role in removing metabolites and misfolded proteins from the brain by facilitating the exchange of para-arterial cerebrospinal fluid (CSF) and paravenous interstitial fluid (ISF) through aquaporin-4 located at astrocytic end feet (Iliff et al., 2012; Christensen et al., 2022). While the gold standard for evaluating GS function involves invasive intrathecal contrast agent injection and repeated MRI scans (Eide and Ringstad, 2015), a novel non-invasive method, DTI-ALPS, has been developed and demonstrated to be closely correlate with the gold standard (Zhang W. et al., 2021), where decreased DTI-ALPS index indicates impaired GS function (Taoka et al., 2017; McKnight et al., 2021). In this research, we found that the ipsilateral DTI-ALPS index was lower than the contralateral side, consistent with previous studies in TLE (Zhang et al., 2023; Zhao et al., 2023), which seems to indicate the worse glymphatic clearance function in the affected hemisphere. The mechanism may be that excessive oxidative stress, glutamic acid, and pro-inflammatory mediators produced by seizures can induce disruption of the blood–brain barrier and may lead to abnormalities in the CSF-ISF circulation, thereby leading to GS dysfunction (Gorter et al., 2015; Liu et al., 2020). The potential vulnerability of the ipsilateral hemisphere of patients with unilateral focal epilepsy (Sidhu et al., 2018) and the independent blood supply system of the bilateral hemispheres (Zhang et al., 2023) may contribute to asynchronous GS function changes.

However, some controversies regarding the interpretation of the DTI-ALPS method have recently been raised (Agarwal et al., 2024; Ringstad, 2024). The main arguments are as follows: (1) Due to the partial volume effect and the limited imaging resolution, the ROI may contain information from the nonperivascular space in addition to the perivascular space (PVS) (Ringstad, 2024). (2) Based on prior findings that blood vessels are more abundant in the cortex than in the subcortical white matter, along with the sparse tracer enhancement observed in deep white matter areas, Ringstad (2024) supposed that the GS plays a minor role in brain clearance within the deep white matter. The above experimental results only indicate that the number of PVS in deep white matter is less than that in the cortex, but cannot infer that the role of GS in clearing metabolic waste in deep white matter is smaller and Ringstad’s speculation is lack of pathophysiological confirmation. In 2021, Zhang et al. placed ROIs in six brain areas (lateral ventricle, third ventricle, fourth ventricle, precentral gyrus, frontal horn, and inferior frontal gyrus) on 3D-T1WI at baseline and 39 h after intrathecal gadolinium injection to obtain glymphatic clearance function (Zhang W. et al., 2021). They found that the DTI-ALPS index was always correlated with glymphatic clearance function calculated by the gold standard in all six brain regions and thus concluded that the DTI-ALPS index might represent glymphatic clearance function. Most studies have found that the DTI-ALPS index is associated with other GS evaluation parameters, such as PVS volume and CPV (Tu et al., 2023; Pang et al., 2024). Regardless of the specific mechanism underlying this index, we believe that the correlation between the decrease in its value and the decline in GS function does exist. Therefore, we propose that the DTI-ALPS index can serve as an indirect imaging marker of GS function.

No significant difference was observed between ipsilateral and contralateral CPV in unilateral TLE patients in our study. This lack of difference may be attributed to the communication of CSF between the lateral ventricles through the interventricular foramen, which exposes the CP on both sides to the same fluid pressure within the lateral ventricles (Lehtinen et al., 2013). Currently, most previous studies have investigated the role of total CPV rather than the unilateral CPV (Choi et al., 2022; Jeong et al., 2023; Jiang et al., 2023b). In a physiologically normal state, CP, a highly vascularized tissue, plays a crucial role in regulating CSF production (Lindvall et al., 1978; Moskowitz et al., 1979; Lindvall and Owman, 1981). CP regulates GS function through several possible mechanisms. First, CP may respond to increased metabolic waste by promoting CSF production, thereby facilitating the removal of waste products in the brain (McKnight et al., 2020). Second, the clearance function of GS is associated with the sleep–wake cycle, with GS function being more active at night (Xie et al., 2013). The synchronization of CSF production and drainage improves the effectiveness of waste elimination, suggesting that clearance efficiency is optimized when circadian rhythms of CSF production align with GS activity (Myung et al., 2018). Enlarged CP, associated with factors such as increased blood-CSF barrier permeability and oxidative stress, has been confirmed to correlate with impaired glymphatic clearance function (Li et al., 2023). In this study, both ipsilateral and contralateral DTI-ALPS index, as well as the mean DTI-ALPS index, exhibited a moderately negative correlation with CP enlargement, probably indicating an association between enlarged CP and impaired glymphatic clearance function in TLE patients. The CP-associated GS dysfunction could be attributed to the impaired astrocyte-dependent CSF-ISF exchange caused by inflammatory chemicals in the CSF (Xie et al., 2024). In addition, our correlation analyses in HCs revealed that CPV was negatively correlated with the MMSE score, a finding also observed in the Alzheimer’s continuum (Jiang et al., 2024), cerebral small vessel disease (Zhang W. et al., 2021) and older adults with objectively normal cognition (Park et al., 2023). To confirm this conclusion, however, future studies with large, healthy populations are needed.

The SVF test is a widely used cognitive screening tool that assesses language generation speed, naming ability, memory, executive function, semantic organization, and extraction tactics (Lezak et al., 2012). Impairment in semantic memory can also affect other cognitive functions (Mardh et al., 2013). In this study, we assessed two aspects of verbal fluency, phonological fluency and semantic fluency. Our findings indicated that GS function correlated with SVF but not with PFT. This observation is partially supported by previous research showing that patients with unilateral temporal lobe lesions had impaired semantic fluency with maintaining intact phonological fluency (Troyer et al., 1998). These findings can be explained by the anatomical and functional characteristics of the temporal lobe. Anatomically, the bilateral anterior temporal lobe is identified as the semantic hub, an area affected in cases of semantic dementia (Hodges and Patterson, 2007; Patterson et al., 2007; Landin-Romero et al., 2016). In neurologically normal participants, the left temporal lobe density exhibited a stronger correlation with SVF than with PFT (Grogan et al., 2009). Functionally, the left temporal cortex demonstrated greater activation during semantic fluency than phonological fluency (Gourovitch et al., 2000).

Recent research has demonstrated semantic memory impairment in TLE patients (Giovagnoli et al., 2005; Messas et al., 2008; Lomlomdjian et al., 2011), which was also observed in our study. Our findings of associations between glymphatic function and semantic fluency performance are partially supported by a study that found enlarged perivascular space (EPVS) burden was associated with worse semantic memory performance (Javierre-Petit et al., 2020). EPVS is considered a potential indicator of decreased glymphatic clearance (Braffman et al., 1988; Wardlaw et al., 2013), which could lead to the accumulation of neurotoxic metabolites (Hlauschek et al., 2024). Previous research in early dementia mice has shown that unimpeded GS function plays an important role in maintaining memory functions (Lee et al., 2020). To the best of our knowledge, this study is the first to demonstrate that GS dysfunction exacerbates the semantic fluency impairment in TLE patients, but further research is needed to corroborate this finding by integrating various MRI methods of evaluating human GS function, such as free water analysis and PVS volume, to have a more comprehensive understanding of the role of GS in semantic fluency performance (Taoka et al., 2024).

To examine the clinical relevance of CP enlargement, we analyzed the association between CP enlargement and semantic fluency performance. An indirect association totally mediated by the mean DTI-ALPS index of bilateral hemispheres was identified. Additionally, the relationship between CP enlargement in the ipsilateral (or contralateral) hemisphere and semantic fluency performance was found to be mediated by the DTI-ALPS index in the ipsilateral (or contralateral) hemisphere. As far as we are aware, this is the first research demonstrating the mediating role of the DTI-ALPS index in the relationship between CP enlargement and semantic fluency performance. Previous studies in multiple sclerosis, where the incidence of epilepsy is 2.5 times than that of normal people (Burman and Zelano, 2017), have reported that the relationship between CP enlargement and deep gray matter atrophy was partially mediated by the DTI-ALPS index (Xie et al., 2024), and that deep gray matter volume was positively correlated with semantic fluency (Kania et al., 2024). We hypothesize that a similar mediating path occurs in TLE. Specifically, aberrant glymphatic drainage caused by abnormal CSF production could lead to changes in deep grey matter volume, which may further contribute to impaired semantic fluency performance. The underlying mechanism could be either that the rhythm of CP production is impacted by epileptic seizure and the nonsynchronous state between the circadian rhythm of CP production and GS activity results in poor GS function, or that the reduced capacity to produce more CSF in response to increased metabolic waste affects fluid flow within the GS, thereby reducing its clearance. The accumulation of metabolic waste in the brain caused by the deterioration of GS function may be the cause of the neuronal damage that may lead to semantic fluency impairment. However, this needs confirmation in future studies with large sample sizes and complex experimental designs where deep grey matter volume is available. Our mediation analyses suggest that the impaired clearance function indicated by the DTI-ALPS index is more closely related to SVF performance than the CSF production function represented by CP. Future insights into how CP function and SVF relate to each other as well as which part of GS function is more strongly correlated with semantic fluency performance are needed. To improve semantic fluency in TLE patients, promoting CSF drainage rather than production may be a preferable therapeutic target.

Our study has several limitations. First, the generalizability of our findings is constrained by this being a single-center study with a small sample size. Second, the relationship between glymphatic measures and neuropsychological performance did not account for potential confounders, such as medication use. Third, we did not explore the role of the burden of EPVS, a known marker of dementia and cognitive deterioration (Yang et al., 2023), in neuropsychological performance. Fourth, as the participants exhibited relatively high cognitive function, it remains unknown whether GS involvement in semantic fluency is present in epilepsy patients with cognitive impairment or dementia. Finally, the causal relationship between CP enlargement and decreased DTI-ALPS index cannot be fully deduced from this cross-sectional study. Large-scale longitudinal studies are needed to validate this proposed mediation relationship.

## 5 Conclusion

Our study provides evidence of decreased DTI-ALPS index in the ipsilateral hemisphere compared to the contralateral side in unilateral TLE. Decreased DTI-ALPS index and increased CPV may be the independent risk factor for semantic fluency impairment. The DTI-ALPS index may fully mediate the relationship between CP enlargement and semantic fluency performance. These findings establish a radiological basis for future investigations into the role of the GS in the pathophysiology of TLE.

## Data Availability

The raw data supporting the conclusions of this article will be made available by the authors, without undue reservation.
