# Supplementary material for: Association of glymphatic system dysfunction with cognitive impairment in temporal lobe epilepsy
Source: Front Aging Neurosci. 2024 Oct 18;16:1459580. doi: 10.3389/fnagi.2024.1459580 (PMC11527717; doi:10.3389/fnagi.2024.1459580)
Supplement: Supplementary file 1 [file Data_Sheet_1.docx]

**
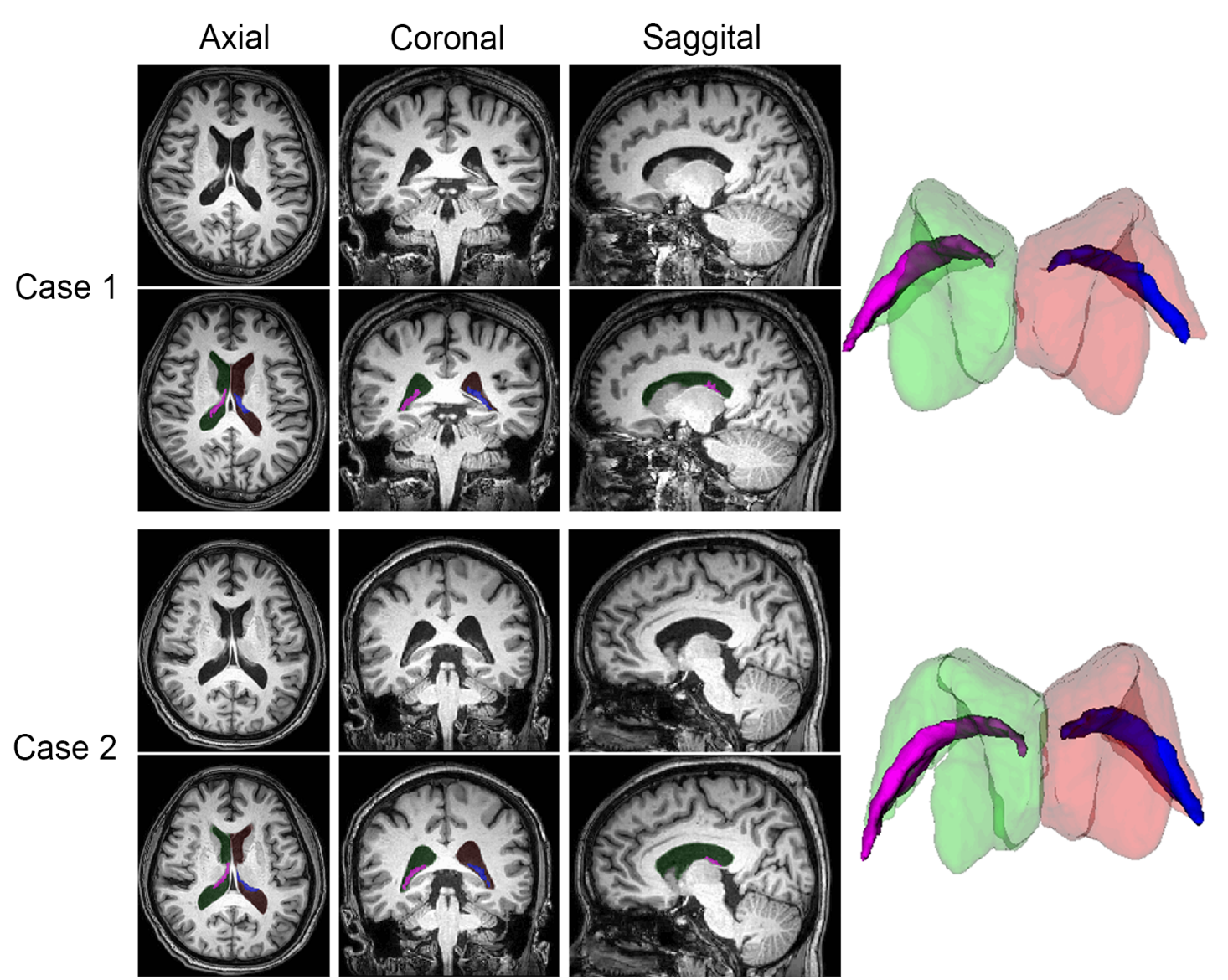
**

**Supplementary Figure S1** **Two cases of VB-Nets model-based CP segmentation.** CP volume is segmented from 3D T1-weighted images according to the Desikan-Killiany atlas using a pre-trained cascaded VB-Nets model integrated into the uRP tool from the axial, coronal and, sagittal points of view. Abbreviations: CP, choroid plexus; 3D, three-dimensional.
