# Supplementary material for: Association of glymphatic system dysfunction with cognitive impairment in temporal lobe epilepsy
Source: Front Aging Neurosci. 2024 Oct 18;16:1459580. doi: 10.3389/fnagi.2024.1459580 (PMC11527717; doi:10.3389/fnagi.2024.1459580)
Supplement: Supplementary file 2 [file Data_Sheet_2.docx]

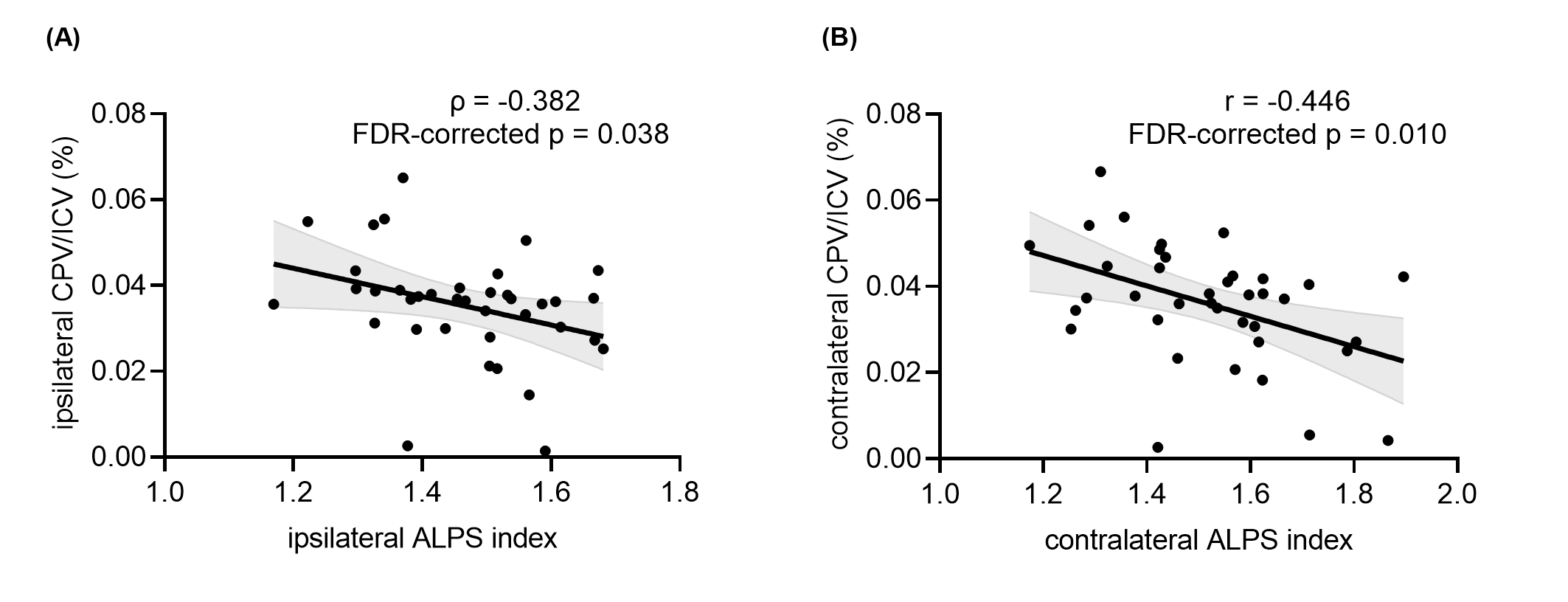


**Supplementary Figure S2 Correlation analyses between ipsilateral (or contralateral) DTI-ALPS index and ipsilateral (or contralateral) CPV/ICV in all TLE patients**

Correlation between ipsilateral ALPS index and ipsilateral CPV/ICV (**A**), contralateral ALPS index and contralateral CPV/ICV (**B**) in patients with unilateral TLE. r and ρ represent Pearson’s correlation coefficient and Spearman’s correlation coefficient, respectively.

Abbreviations: DTI-ALPS, diffusion tensor image analysis along the perivascular space; CPV, choroid plexus volume; ICV, intracranial volume; TLE, temporal lobe epilepsy; FDR, false discovery rate.
