# Supplementary material for: Association of glymphatic system dysfunction with cognitive impairment in temporal lobe epilepsy
Source: Front Aging Neurosci. 2024 Oct 18;16:1459580. doi: 10.3389/fnagi.2024.1459580 (PMC11527717; doi:10.3389/fnagi.2024.1459580)
Supplement: Supplementary file 3 [file Table_1.docx]

**Supplementary Table S1. Correlation between the DTI-ALPS index, CPV and neuropsychological performance in all TLE patients**

|  | **Ipsilateral**  **DTI-ALPS index** | | **Contralateral**  **DTI-ALPS index** | | **Mean**  **DTI-ALPS index** | | **CPV/ICV (%)** | | **Ipsilateral CPV/ICV (%)** | | **Contralateral**  **CPV/ICV (%)** | |
| --- | --- | --- | --- | --- | --- | --- | --- | --- | --- | --- | --- | --- |
|  | **r /ρ** | **p value** | **r /ρ** | **p value** | **r /ρ** | **p value** | **r /ρ** | **p value** | **r /ρ** | **p value** | **r /ρ** | **p value** |
| MoCA | 0.208 | 0.209 | 0.306 | 0.061 | 0.274 | 0.096 | -0.315 | 0.054 | -0.321 | 0.050 | -0.220 | 0.184 |
| MMSE | 0.193 | 0.247 | 0.139 | 0.406 | 0.171 | 0.304 | -0.168 | 0.313 | -0.208 | 0.209 | -0.087 | 0.604 |
| AT | 0.313 | 0.055 | 0.318 | 0.052 | 0.339 | 0.037* | -0.400 | 0.013* | -0.357 | 0.028* | -0.354 | 0.029* |
| DSST | 0.249 | 0.132 | 0.303 | 0.065 | 0.300 | 0.067 | -0.380 | 0.019* | -0.334 | 0.040* | -0.316 | 0.053 |
| DST | 0.315 | 0.058 | 0.385 | 0.018* | 0.382 | 0.020* | -0.280 | 0.093 | -0.213 | 0.206 | -0.243 | 0.147 |
| BNT | 0.173 | 0.307 | 0.351 | 0.033* | 0.301 | 0.070 | -0.299 | 0.073 | -0.361 | 0.028* | -0.183 | 0.278 |
| Block design | 0.185 | 0.274 | 0.210 | 0.211 | 0.238 | 0.156 | -0.258 | 0.123 | -0.315 | 0.058 | -0.198 | 0.241 |
| PFT | 0.095 | 0.568 | 0.161 | 0.333 | 0.143 | 0.391 | -0.174 | 0.295 | -0.152 | 0.362 | -0.163 | 0.327 |
| SVF | 0.477 | 0.0049** | 0.482 | 0.002** | 0.502 | 0.001** | -0.393 | 0.015* | -0.501 | 0.001** | -0.362 | 0.026* |

* indicates statistically significant p < 0.05. ** indicates statistically significant p < 0.05 after FDR correction.

r and **ρ** represent Pearson’s correlation coefficient and Spearman’s rank correlation coefficient, respectively.

Abbreviations: MoCA, Montreal Cognitive Assessment; MMSE, Minimum Mental State Examination, AT, Arithmetic Test; DSST, Digit Symbol Substitution Test; DST, Digital Span Test; BNT, Boston Naming Test; PFT, Phonological Fluency Test; SVF, Semantic Verbal Fluency; DTI-ALPS, diffusion tensor image analysis along the perivascular space; CPV, choroid plexus volume; ICV, intracranial volume; FDR, false discovery rate. The uncorrected p values are shown in the table.
