# Supplementary material for: Association of glymphatic system dysfunction with cognitive impairment in temporal lobe epilepsy
Source: Front Aging Neurosci. 2024 Oct 18;16:1459580. doi: 10.3389/fnagi.2024.1459580 (PMC11527717; doi:10.3389/fnagi.2024.1459580)
Supplement: Supplementary file 4 [file Table_2.docx]

**Supplementary Table S2. Correlation between the mean DTI-ALPS index, CPV and neuropsychological performance in HCs**

|  | **the mean DTI-ALPS index** | | **CPV/ICV (%)** | |
| --- | --- | --- | --- | --- |
|  | **r/ρ** | **p value** | **r/ρ** | **p value** |
| MoCA | 0.182 | 0.384 | -0.233 | 0.252 |
| MMSE | 0.032 | 0.880 | -0.552 | 0.003** |
| AT | 0.181 | 0.387 | -0.198 | 0.331 |
| DSST | -0.375 | 0.065 | 0.022 | 0.914 |
| DST | -0.064 | 0.762 | -0.006 | 0.978 |
| BNT | -0.049 | 0.816 | 0.319 | 0.112 |
| Block design | -0.255 | 0.229 | 0.432 | 0.031* |
| PFT | -0.291 | 0.158 | -0.135 | 0.510 |
| SVF | -0.135 | 0.519 | 0.078 | 0.703 |

* indicates statistically significant p < 0.05. ** indicates statistically significant p < 0.05 after FDR correction. r and **ρ** represent Pearson’s correlation coefficient and Spearman’s rank correlation coefficient, respectively.

Abbreviations: MoCA, Montreal Cognitive Assessment; MMSE, Minimum Mental State Examination, AT, Arithmetic Test; DSST, Digit Symbol Substitution Test; DST, Digital Span Test; BNT, Boston Naming Test; PFT, Phonological Fluency Test; SVF, Semantic Verbal Fluency; DTI-ALPS, diffusion tensor image analysis along the perivascular space; CPV, choroid plexus volume; ICV, intracranial volume; FDR, false discovery rate; HCs, healthy controls. The uncorrected p values are shown in the table.
