# Supplementary material for: Association of glymphatic system dysfunction with cognitive impairment in temporal lobe epilepsy
Source: Front Aging Neurosci. 2024 Oct 18;16:1459580. doi: 10.3389/fnagi.2024.1459580 (PMC11527717; doi:10.3389/fnagi.2024.1459580)
Supplement: Supplementary file 5 [file Table_3.docx]

**Supplementary Table S3. Final results of multivariable linear regression (backward stepwise) to explore the role of ipsilateral or contralateral CPV in SVF performance in the TLE group**

|  |  | **B** | **Beta coefficient (β)** | **p value** | **Adjust-R^2^** |
| --- | --- | --- | --- | --- | --- |
| Model 1 | Educational level^*^ |  | | | 0.232 |
|  | college or equivalent | 2.930 | 0.357 | 0.020 |  |
|  | Ipsilateral CPV/ICV (%) | -105.835 | -0.323 | 0.034 |  |
| Model 2 | Educational level^*^ |  | | | 0.219 |
|  | college or equivalent | 3.003 | 0.366 | 0.018 |  |
|  | Contralateral CPV/ICV (%) | -90.749 | -0.302 | 0.048 |  |

The initial regression model included the following six variables: age, sex, education level (divided into less than college and college or equivalent), disease duration, seizure frequency (divided into 0-11/Year and ≥11/Year), ipsilateral CPV/ICV (**Model 1**) or contralateral CPV/ICV (**Model 2**). Using the backward elimination method, **Model 1** and **Model 2** were ultimately constructed by the above variables in the table, which indicated that increased ipsilateral CPV/ICV and contralateral CPV/ICV were both independent risk factors for semantic fluency impairment. This table shows the variables entered into the final model after backward elimination. Variables not included in the final models (age, sex, disease duration and seizure frequency) are not shown.

^*^ Reference category: less than college

Abbreviations: SVF, Semantic Verbal Fluency; TLE, Temporal lobe epilepsy; CPV, choroid plexus volume; ICV, intracranial volume.
